# 20(S)-protopanaxadiol inhibits proliferation and induces apoptosis of acute myeloid leukemia cells via targeting Bcl-X_L_ and MCL-1

**DOI:** 10.3389/fphar.2025.1530270

**Published:** 2025-04-29

**Authors:** Meng Xu, Feng Hao, Shuangshuang Wu, Zhanning Qu, Junying Li, Shuang Chen, Fang Fang, Yundong Zhao, Cheng Hu

**Affiliations:** ^1^ College of Laboratory Medicine, Jilin Medical University, Jilin City, Jilin, China; ^2^ School of Medical Technology, Beihua University, Jilin City, Jilin, China; ^3^ Medical Laboratory Department, Hepatobiliary Hospital of Jilin Province, Changchun, Jilin, China; ^4^ Department of Pediatric Hematology, Children’s Medical Center, The First Hospital of Jilin University, Changchun, Jilin, China; ^5^ Second Affiliated Hospital of Guangdong Medical University, Guangzhou, Guangdong, China

**Keywords:** acute myeloid leukemia, 20(S)-protopanaxadiol, MCL-1, Bcl-X_L_, apoptosis

## Abstract

Currently, intensive chemotherapy with cytarabine and anthracycline (the “7 + 3” regimen) and hypomethylating agents remains the standard treatment for patients with acute myeloid leukemia (AML). Despite advances in treatment such as targeted therapies, patient outcomes remain unsatisfactory due to adverse drug reactions, susceptibility to drug resistance, and high recurrence rates. Consequently, there is an urgent need to develop safer and more efficacious treatments for AML. In this study, we examined the effects of 20(S)-protopanaxadiol (20(S)-PPD) on AML cells. Our findings indicate that 20(S)-PPD inhibits cell proliferation and induces apoptosis in AML cells. Mechanistically, 20(S)-PPD-induced apoptosis was at least partially dependent on the anti-apoptotic proteins MCL-1 and Bcl-X_L_. Moreover, the downregulation of MCL-1 and Bcl-X_L_ by 20(S)-PPD is mediated through the inhibition of transcription and a decrease in protein stability. Additionally, results from virtual molecular docking demonstrated that 20(S)-PPD exhibits lower binding energies with MCL-1 and Bcl-X_L_ (−7.58 and −8.75 kcal/mol, respectively), suggesting that 20(S)-PPD may directly interact with these proteins, thereby accelerating their degradation. Finally, 20(S)-PPD has been shown to synergistically enhance the anti-leukemic activity of venetoclax, a selective Bcl-2 inhibitor, in AML cells. The current study suggests that the continued development of 20(S)-PPD as a therapeutic agent for AML would be advantageous.

## 1 Introduction

Acute myeloid leukemia (AML) occurs when immature myeloid progenitor cells accumulate in the bone marrow and peripheral blood due to impaired clonal proliferation and differentiation (Siveen et al., 2017). According to estimates, AML has a 5-year overall survival rate of only 30% and is considered the most lethal subtype of acute leukemia (Khwaja et al., 2016; Lai et al., 2019; Shimony et al., 2023). The intensive induction chemotherapy regimen, known as the “7 + 3 regimen” and consisting of cytarabine and anthracyclines, has been a mainstay in the therapeutic management of AML since 1973 (Lichtman, 2013). However, this intensive chemotherapy is not suitable for elderly patients or those with adverse risk cytogenetics or comorbidities. Although pediatric AML patients have fewer comorbidities and can tolerate intensive treatment better than adults, the overall survival rate (approximately 65%) is still not satisfactory. Advances in molecular technologies have revealed numerous molecular abnormalities that contribute to AML pathogenesis (Lai et al., 2019; Nwosu et al., 2024). Consequently, targeted therapies have been developed and recently approved for the treatment of AML, including agents that target FMS-like tyrosine kinase 3 (FLT3), isocitrate dehydrogenase (IDH), and Bcl-2 (Shimada, 2019). Nonetheless, new clinical challenges have emerged due to the development of resistance to these agents (Qi et al., 2015; Wei and Roberts, 2023). Therefore, it is essential to develop more effective or adjuvant therapies for AML with urgency.

Over recent decades, numerous active molecules derived from traditional Chinese medicinal plants have shown efficacy in the prevention and treatment of cancer, exhibiting lower toxicity and fewer side effects, thereby representing a promising therapeutic option (Zhang et al., 2021). Ginsenosides, a class of triterpenoid saponins extracted from ginseng roots, constitute the primary pharmacologically active constituents of ginseng (Shi et al., 2019). Saponins in ginseng are primarily classified into three types: dammarane-type saponins (such as protopanaxadiol and protopanaxatriol), ocotillol-type saponins, and oleanane-type saponins, with the dammarane type being the most prevalent (Wong et al., 2015; Liu et al., 2020).

20(S)-protopanaxadiol (20(S)-PPD) is produced through acid hydrolysis and the action of intestinal bacteria via a series of de-glycosylation processes after oral administration of ginsenosides. This transformation enhances their bioavailability and facilitates absorption by the human body (Jo et al., 2021). Studies have revealed that 20(S)-PPD is an effective antitumor agent against various solid tumors, demonstrating its efficacy both *in vitro* and *in vivo* (Zhu et al., 2011; Zhang et al., 2013; Ben-Eltriki et al., 2016; Zhang et al., 2018; Peng et al., 2019; Lu et al., 2020; Zhang et al., 2020; Fu et al., 2022; Song et al., 2023). Given the paucity of data on AML, we conducted an investigation into the cytotoxic effects and underlying mechanisms of 20(S)-PPD on AML cells. Here, we show that 20(S)-PPD inhibits cell proliferation and induces apoptosis in AML cells. Bcl-X_L_ and MCL-1 play crucial roles in 20(S)-PPD-induced apoptosis. Furthermore, the combination of 20(S)-PPD and venetoclax exhibited a synergistic effect in inhibiting proliferation and inducing apoptosis in AML cells.

## 2 Materials and methods

### 2.1 Drugs

20(S)-protopanaxadiol (20(S)-PPD), venetoclax, A-1155463 Dihydrochloride, A-1210477, MG132, and cycloheximide were obtained from the Selleck Chemical (Houston, United States).

### 2.2 Cells lines and culture

THP-1 and MOLM-13 cells were provided by Procell Life Science (Wuhan, China), while MV4-11 cells were obtained from ATCC (Manassas, VA, United States). The cell lines were maintained at 37°C in an incubator with 5% CO_2_ and RPMI-1640 medium supplemented with 100 U/mL penicillin, 100 μg/mL streptomycin, and 10% fetal bovine serum (FBS, CLARK Bioscience, United States). The same conditions were used for THP-1 shRNA knockdown cell lines, but puromycin was added at 2 μg/mL.

### 2.3 CCK-8 assays

The Cell Counting Kit-8 (CCK-8; BioSS, Beijing, China) was used to assess cell viability. Following a 48-h exposure of AML cells to varying concentrations of 20(S)-PPD, 10 μL of the CCK-8 reagent was added to each well of a microplate. Subsequently, the cells were incubated for an additional 2 h to allow for the colorimetric reaction to develop. The optical density (OD) at 450 nm of the cells was measured using a microplate reader (BMG LABTECH, GmbH, Germany).

### 2.4 Annexin V/propidium iodide staining

The Annexin V-FITC/propidium iodide (PI) staining (Bestbio, Shanghai, China) was used to determine cell apoptosis. Briefly, a final concentration of 0, 20, 40, 60 μM of 20(S)-PPD was applied to cells plated into 24-well plates. After treating the cells with 20(S)-PPD at different concentrations, a 5-min centrifugation at 1,500 rpm was performed. The cells were then resuspended in 50 μL of 1× binding buffer, and 3 μL of propidium iodide (PI) and 6 μL of Annexin V-FITC solution were added. The mixtures were incubated in the dark for 15 min at room temperature. Flow cytometry analysis (Beckman, United States) was performed after adding 400 μL of binding buffer to each tube. Based on a typical experiment, the average percentages of Annexin V+/PI- (early apoptosis) and Annexin V+/PI+ (late apoptosis or dead cells) were determined.

### 2.5 Mitochondria membrane potential assay

The JC-10 staining kit (Bestbio, Shanghai, China) was used to assessed mitochondrial membrane potential (MMP). Briefly, AML cells were exposed to 20(S)-PPD at final concentrations of 0, 20, 40, and 60 μM for 8 h. Following treatment, the cells were washed twice with phosphate-buffered saline (PBS) and subsequently centrifuged at 1,500 rpm for 5 min to collect the cells. The cells were then resuspended in 500 μL of JC-10 staining working solution, prepared according to the manufacturer’s instructions, and incubated at 37°C with 5% CO_2_ for 30 min. A reduction in MMP is recognized as a hallmark event in the early stages of apoptosis. When the MMP is high, JC-10 preferentially accumulates within the mitochondrial matrix, resulting in the formation of red fluorescence. In contrast, a low MMP leads to the dissociation of JC-10 into monomers, which emit green fluorescence. Subsequently, flow cytometry analysis was conducted to quantify the fluorescence intensity at excitation/emission wavelengths of 510 nm/570 nm for red fluorescence and 488 nm/520 nm for green fluorescence. The ratio of red to green fluorescence intensity serves as an indicator of the cellular MMP, allowing for normalization of the data.

### 2.6 Western blot analysis

After treating AML cells with 20(S)-PPD at final concentrations ranging from 0 to 60 μM for 8 h, Western blot was performed to detect PARP cleavage and apoptosis-associated proteins. Incubation of cells at 37°C resulted in their harvesting and lysis in ultrasonic lysates containing inhibitors of proteases and phosphatases. The cells were then ultrasonically broken using an ultrasonic crusher with 130 W power, ultrasonication for 1 s, stopping for 1 s, and a total ultrasonication time of 1 min. After centrifuging the total lysate for 10 min at 12,000 rpm, the supernatants were analyzed using a BCA protein assay kit (Thermo Fisher Scientific, United States). We added 20 μg of cell extract to a polyacrylamide-SDS gel containing 5% concentrated gel and 10% separating gel. Once the gels were electrophoretically transferred to PVDF membranes, the membranes were blocked with 5% skim milk for 1 h at room temperature. The membrane, after transfer, was incubated overnight at 4°C with either anti-Bcl-2 family (selleck) or anti-PARP (proteintech) antibodies, both diluted to 1:1,000. After 1 h of incubation with HRP-conjugated secondary antibodies, chemiluminescence (Fine-Do X3, Bioscience, Beijing, China) was used to visualize protein bands using the HRP reaction with its substrates.

### 2.7 Real-time quantitative PCR analysis

Real-time quantitative PCR (qRT-PCR) was used to detect mRNA expression following an 8-h treatment with 20(S)-PPD at concentrations of 0, 20, 40, and 60 μM. Total RNA was isolated using the Total RNA Extraction Kit (Solarbio, China), and cDNA was synthesized using the SureScriptTM First-Strand cDNA Synthesis Kit (GeneCopoeia, China). The MonAmpTM qPCR Kit (Monad Biology, China) and 7,500 Real-Time PCR System (Applied Biosystems, United States) were used in a 20 μL total volume for RT-qPCR. Using the comparative Ct method with β-actin as the housekeeping gene, the 2^−ΔΔCt^ values were calculated to evaluate mRNA expression levels. The primers used for amplification were as follows: MCL-1:5′-GGAGTTGGTCGGGGAATCTG-3′ and 5′-CTC​CCG​AAG​GTA​CCG​AGA​GA-3′; Bcl-X_L_: 5′-AAC​TCT​TCC​GGG​ATG​GGG​TA-3′ and 5′-CTG​CGA​TCC​GAC​TCA​CCA​AT-3′; β-actin: 5′ TCC​TCC​CTG​GAG​AAG​AGC​TAC-3′ and 5′-TCC​TGC​TTG​CTG​ATC​CAC​AT-3′.

### 2.8 Production of lentivirus particles and infection of THP-1 cells

Sigma-Aldrich provided the non-target control vector and Bax-/Bak-shRNA constructs, while Dharmacon supplied the Bcl-X_L_ and MCL-1 open reading frame (ORF) constructs. Lentivirus production was achieved by co-transfecting HEK293T cells with the lentiviral shRNA, pMD-VSV-G, and delta 8.2 plasmids. Viral supernatants were harvested 48 h post-co-transfection, and 1 mL of viral supernatant mixed with 4 μg/mL polybrene (Sigma-Aldrich) was added to THP-1 cells to evaluate infection efficiency. Puromycin at a concentration of 2 μg/mL was used to screen for shRNA lentiviral gene silencing cell lines.

### 2.9 Molecular docking

Using the PubChem database (https://pubchem.ncbi.nlm.nih.gov/), we predicted the 3D structure and binding activity of 20(S)-PPD to the relevant targets. The PDB database (https://www.pdb.org/) was used to download the protein structures of the target proteins and remove ligands and non-protein molecules. Before docking could begin, all hydrogens needed to be added to small-molecule drugs and protein structures. With the help of Pymol software, we visualized the docking results using Auto Dock 4. In docking analyses, the model with the smallest G score was regarded as the most appropriate. Docking of 20(S)-PPD with target proteins required binding energies of at least −5.0 kcal/mol. Only results with these energies were accepted.

### 2.10 Statistical analysis

Statistical analysis was conducted using GraphPad Prism 9.0. Values are presented as mean ± SEM (error bar). Statistical significance was determined with an unpaired two-tailed t-test. The significance threshold was set at P ≤ 0.05 for the column graphs. The IC_50_ was calculated in non-linear regression by selecting the log (inhibitor) vs normalized response - Variable slope model in the Dose-response-Inhibition category.

## 3 Results

### 3.1 20(S)-PPD treatment inhibited proliferation and induced apoptosis in AML cells

**FIGURE 1:**
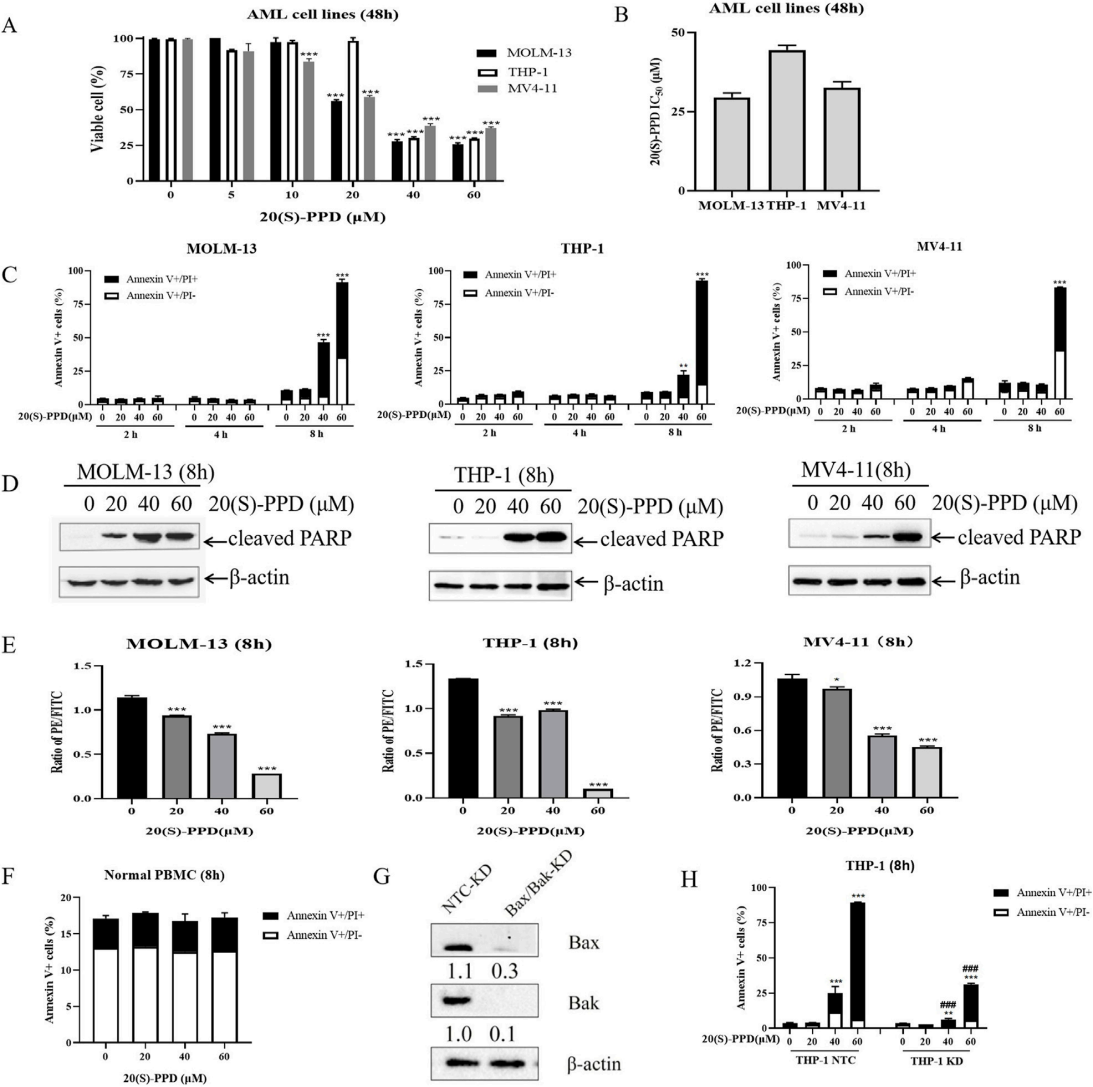
20(S)-PPD treatment inhibited proliferation and induced apoptosis in AML cells. **(A)** CCK-8 assays were performed on 96-well plates containing 0–60 μM 20(S)-PPD for 48 h, with 100% viable cells considered in the vehicle control well. Averaging three duplicate experiments (with standard errors) was used to determine the data. ***P < 0 0.001 compared to the vehicle control. **(B)** Based on three independent experiments, the IC_50_ values of 20(S)-PPD were calculated and graphed. **(C)** Annexin V/PI staining and flow cytometry analysis were performed on MOLM-13, THP-1 and MV4-11 cells after 0–60 μM 20(S)-PPD treatment for 2, 4, and 8 h. From a representative experiment, apoptosis data is presented as the means of triplicates±standard errors. **P < 0.01; ***P < 0.001 compared to the vehicle control. **(D)** Antibodies targeting cleaved PARP were utilized in Western blot analysis of complete cell lysates. **(E)** JC-10 staining were performed to determine MMP in AML cells after 0–60 μM 20(S)-PPD treatment for 8 h. From a representative experiment, apoptosis data is presented as the means of triplicates±standard errors. *P < 0.05; ***P < 0.001 compared to the vehicle control. **(F)** Normal peripheral blood mononuclear cells (PBMCs) were treated with 0–60 μM 20(S)-PPD for 8 h and stained with annexin V-FITC/PI. **(G, H)** THP-1 cells were exposed to NTC-, Bax-, or Bak-shRNA lentivirus for 4–6 h, followed by washing and a 48-h culture in new media before being treated with 20(S)-PPD. The reduction in Bax or Bak expression was confirmed through Western blot analysis **(G)**. 20(S)-PPD treatment of NTC-shRNA, Bax-shRNA/Bak-shRNA knockdown cells were analyzed by flow cytometry **(H)**. **P < 0.01; ***P < 0.001 compared to the no drug treatment control, while ###P < 0.001 compared to 20(S)-PPD-treated NTC-shRNA cells.

AML cells were treated with concentrations ranging from 0 to 60 μM of 20(S)-PPD for 48 h to investigate its anti-proliferative effect. The CCK-8 assay demonstrated that 20(S)-PPD inhibited cell proliferation in a dose-dependent manner (Figure 1A). The IC_50_ values of 20(S)-PPD for MOLM-13, THP-1, and MV4-11 cells were 29.5 ± 1.4 μM, 44.5 ± 1.5 μM, and 32.5 ± 1.9 μM, respectively (Figure 1B). Further investigation using Annexin V/PI staining and flow cytometry analysis showed that all three tested AML cell lines responded positively to 20(S)-PPD treatment for 8 h, as measured by Annexin V-positive percentage and PARP cleavage (Figures 1C, D). Considering that the decrease in MMP is a hallmark event of early apoptosis, JC-10 staining was performed after treating AML cells with 20(S)-PPD for 8 h to assess the MMP. The results in Figure 1E indicate that 20(S)-PPD reduced MMP in a dose-dependent manner in all tested AML cells. These results demonstrate that AML cells undergo apoptosis in response to 20(S)-PPD. A varying concentration of 20(S)-PPD was then administered for 8 h to peripheral blood mononuclear cells (PBMCs) from healthy donors to evaluate its effect on normal cells. Normal PBMCs treated with 20(S)-PPD did not exhibit an increased percentage of Annexin V-positive cells (Figure 1F), suggesting that 20(S)-PPD may not be toxic to them.

In the mitochondrial apoptotic pathway, Bcl-2 (B Cell Lymphoma 2) proteins Bax and Bak, central effectors of mitochondrial apoptosis, play key roles. When apoptotic signals are initiated, both Bax and Bak insert into the outer mitochondrial membrane, forming oligomers that alter the permeability of the mitochondrial membrane. This change allows the release of cytochrome c, leading to the activation of caspases and ultimately resulting in apoptosis (Shamas-Din et al., 2013). With lentiviral vectors containing Bax/Bak shRNA, we engineered THP-1 cells with a double knockdown of Bax and Bak to determine whether 20(S)-PPD induces apoptosis via the intrinsic/mitochondrial apoptotic pathway. Western blot analysis revealed that, in comparison to the sh-NTC (non-target control), the protein expressions of Bax and Bak were evidently reduced in the Bax/Bak dual knockdown THP-1 cells (Figure 1G). Dual knockdown significantly mitigated 20(S)-PPD-induced apoptosis on AML cells (Figure 1H), indicating that mitochondrial/intrinsic pathways are involved in 20(S)-PPD-induced apoptosis. Based on the data presented in Figure 1, 20(S)-PPD appears to inhibit proliferation and induce apoptosis in AML cell lines, but not in normal PBMCs. The apoptosis induced by 20(S)-PPD appears to be mediated, at least in part, through the intrinsic/mitochondrial apoptotic pathway.

### 3.2 20(S)-PPD treatment downregulated the expression levels of Bcl-X_L_ and MCL-1 in AML cells

**FIGURE 2:**
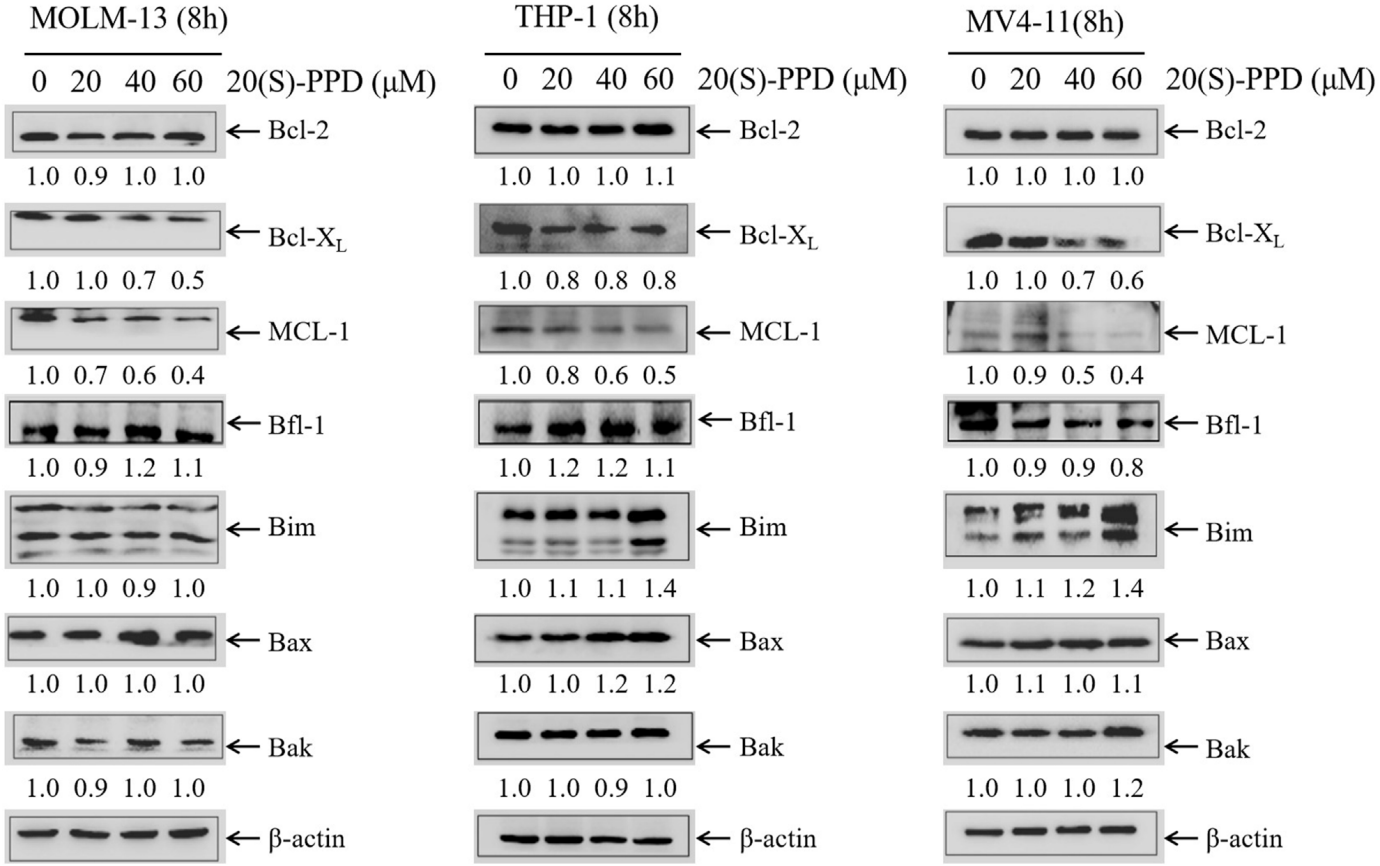
20(S)-PPD treatment downregulated the expression of anti-apoptotic proteins MCL-1 and Bcl-X_L_ in AML cells. After treating MOLM-13, THP-1 and MV4-11 cells for 8 h with 0–60 μM 20(S)-PPD, whole cell lysates were analyzed by Western blotting. Densitometry measurements are shown below the corresponding blots, normalized to β-actin and compared to the vehicle control group.

It is well established that the Bcl-2 family proteins are critical regulators of intrinsic/mitochondrial apoptosis, functioning as both promoters and inhibitors of cell death (Czabotar and Garcia-Saez, 2023). Given that 20(S)-PPD-induced apoptosis involves the intrinsic/mitochondrial pathway, we examined the levels of the anti-apoptotic proteins Bcl-2, Bcl-X_L_, and MCL-1 and the pro-apoptotic proteins Bax, Bak, and Bim using Western blotting in AML cells following increasing concentrations of 20(S)-PPD for 8 h. The data presented in Figure 2 indicate that treatment with 20(S)-PPD resulted in a reduction of MCL-1 and Bcl-X_L_ levels, while the expression of Bcl-2, Bfl-1, Bax, and Bak remained unaffected across all three tested AML cell lines. Additionally, the expression of Bim exhibited a slight increase in THP-1 and MV4-11 cells, but remained unchanged in MOLM-13 cells following 20(S)-PPD treatment. These findings suggest that the downregulation of MCL-1 and Bcl-X_L_ may play a role in mediating 20(S)-PPD-induced apoptosis in AML cells.

### 3.3 Bcl-X_L_ and MCL-1 play roles in 20(S)-PPD-induced apoptosis

**FIGURE 3:**
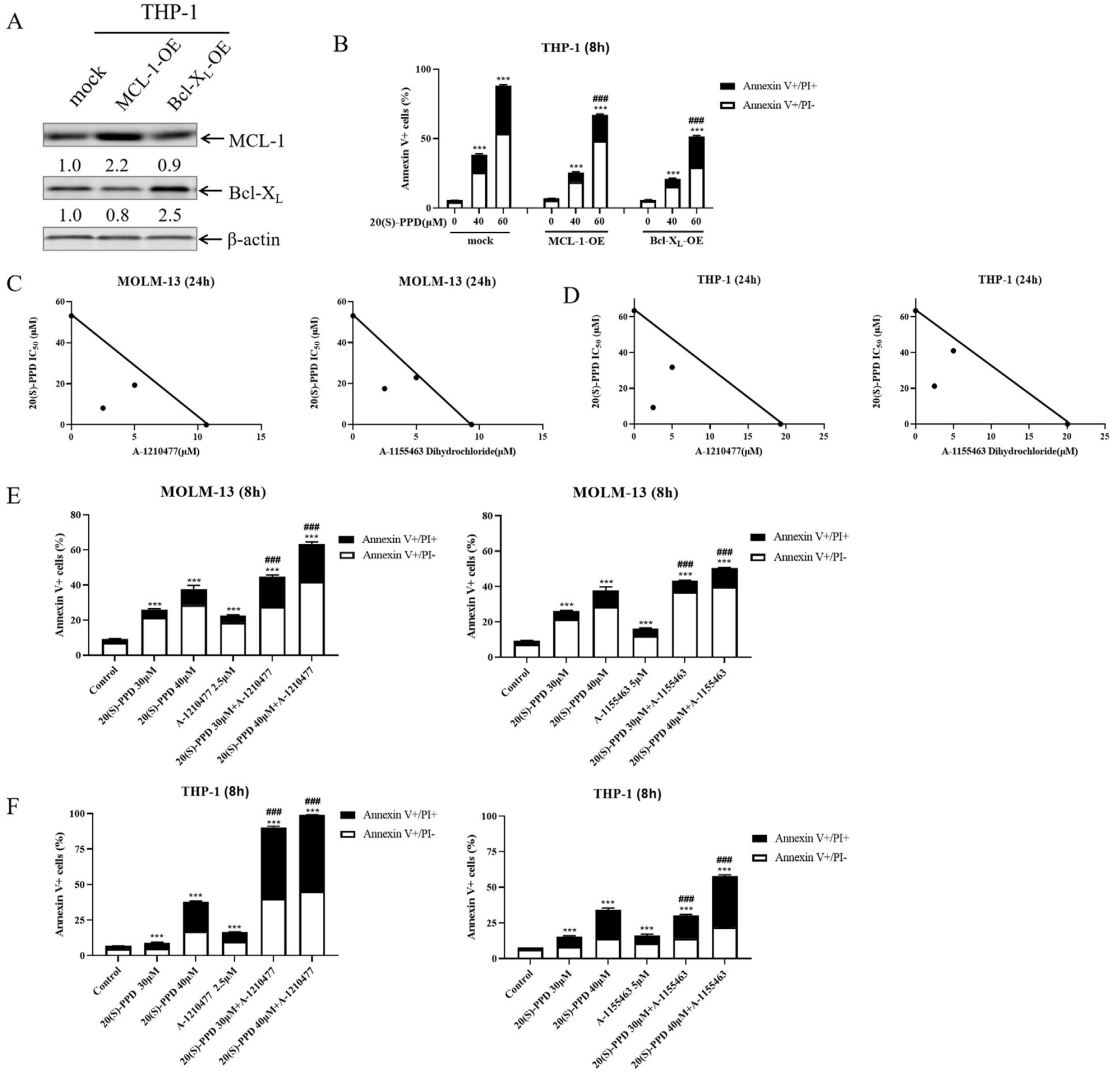
Bcl-X_L_ and MCL-1 play important roles in 20(S)-PPD-induced apoptosis. **(A, B)** Infection of THP-1 cells with Precision LentiORF Bcl-X_L_, MCL-1, and mock (negative control) lentivirus for 4–6 h was followed by washing and incubation in fresh media for 48 h. A whole cell lysate was subjected to Western blot analysis with anti-Bcl-X_L_ or anti-MCL-1 antibodies **(A)** and compared with a mock group to determine density. OE, overexpression. After treating the cells with 0–60 μM 20(S)-PPD for 8 h, Annexin V/PI staining and flow cytometry analyses were performed **(B)**. ***P < 0.001 compared to no drug treatment control, while ###P < 0.001, compared to 20(S)-PPD treated mock group. **(C, D)** Using MOLM-13 cells **(C)** and THP-1 cells **(D)**, we treated them with 20(S)-PPD alone or combined with theA-1210477 (MCL-1 inhibitor) or A-1155463 Dihydrochloride (Bcl-X_L_ inhibitor) for 24 h. After that, we performed a CCK-8 assay and calculated IC_50_ values. The axes show the IC_50_ values for each inhibitor. There is a solid line to indicate additive effects, while the points show concentrations that inhibit proliferation by 50%. A synergistic point is below the line, whereas an antagonistic point is above it. **(E, F)** MOLM-13 cells **(E)** and THP-1 cells **(F)** were treated with 20(S)-PPD alone or in combination with the A-1210477 or A-1155463 Dihydrochloride for 8 h and then stained with Annexin V/PI staining and flow cytometry analyses. ***P < 0.001 compared to the no drug treatment control, while ###P < 0.001 compared to 20(S)-PPD-treated group.

Using lentivirus containing ORFs of Bcl-X_L_ and MCL-1, we successfully overexpressed Bcl-X_L_ and MCL-1 in THP-1 cells to study their involvement in 20(S)-PPD-induced apoptosis (Figure 3A). Figure 3B shows that the overexpression of Bcl-X_L_ and MCL-1 partially mitigated 20(S)-PPD-induced apoptosis as determined by annexin V/PI staining and flow cytometry.

A-1210477 (MCL-1 inhibitor) and A-1155463 Dihydrochloride (Bcl-X_L_ inhibitor), respectively, were co-treated with 20(S)-PPD to further study their roles in 20(S)-PPD-induced apoptosis. As demonstrated in Figures 3C, D, the CCK-8 assay revealed that the inhibitors of MCL-1 or Bcl-X_L_ synergistically inhibited the proliferation of THP-1 and MOLM-13 cells when combined with 20(S)-PPD. Similarly, the MCL-1 or Bcl-X_L_ inhibitors significantly enhanced 20(S)-PPD-induced apoptosis in the two AML cell lines, as illustrated in Figures 3E, F. Collectively, these results indicate that 20(S)-PPD induces apoptosis in AML cells by downregulating the levels of MCL-1 or Bcl-X_L_, as shown in Figure 3.

### 3.4 20(S)-PPD treatment reduced the mRNA level and protein stability of Bcl-X_L_ and MCL-1

**FIGURE 4:**
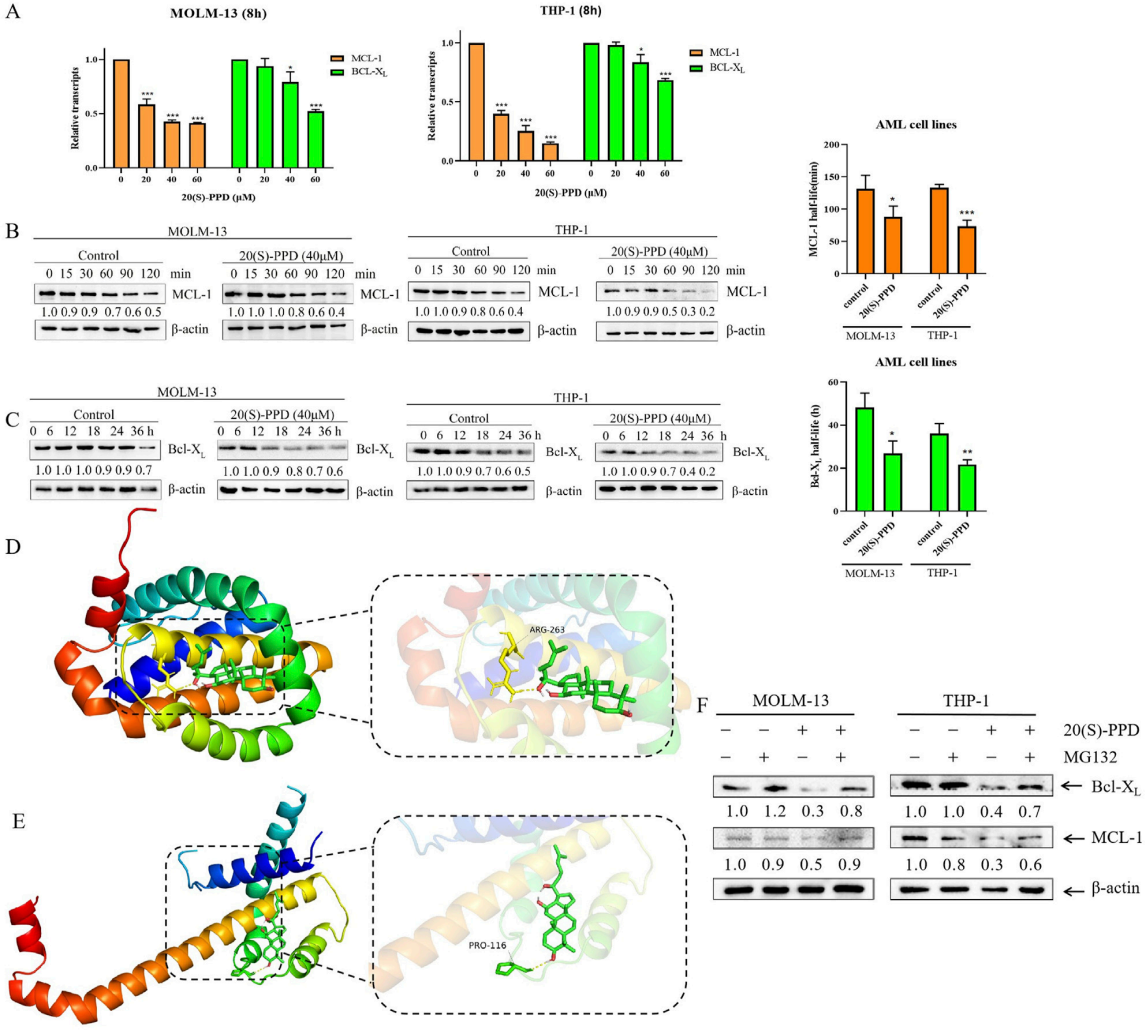
20(S)-PPD treatment decreased the mRNA and protein stability of Bcl-X_L_ and MCL-1. **(A)** We isolated RNA from MOLM-13 and THP-1 cells and detected Bcl-X_L_ and MCL-1 transcript levels using qRT-PCR after treatment with 0–60 μM 20(S)-PPD for 8 h. The fold change normalized to β-actin. Data are represented as means ± SD of at least triplicate measurements. *P < 0.05; ***P < 0.001 compared to the no drug treatment control. **(B, C)** MOLM-13 cells **(B)** and THP-1 cells **(C)** were treated with 40 μM 20(S)-PPD for 8 h, and then washed away the drug and added 10 μg/mL of Cycloheximide (CHX) to inhibit protein synthesis. Western blotting was probed with indicated antibodies. The density measurements for the corresponding blots are shown below, normalized to β-actin. Representative western blots are shown on the left, while half-life time shown on the right. *P < 0.05; **P < 0.01 compared to the no drug treatment control. **(D, E)** The graph of virtual molecular docking. 20(S)-PPD interacts directly with MCL-1 **(D)** and Bcl-X_L_ **(E)**. **(F)** MOLM-13 and THP-1 cells were exposed to 40 μM 20(S)-PPD for 8 h, either alone or in combination with 300 nM MG132, for 24 h. Subsequently, Western blotting was conducted to analyze the levels of Bcl-X_L_ and MCL-1. The resulting band densities were compared to those of the vehicle control and normalized to β-actin.

20(S)-PPD was treated for 8 h in THP-1 and MOLM-13 cells in order to explore the potential molecular mechanisms by which it regulates Bcl-X_L_ and MCL-1 expression. qRT-PCR was performed with actin as the reference gene, and the data were subsequently normalized to these baseline values. The relative quantification was exhibited by calculating the 2^−ΔΔCt^ values. A dose-dependent decrease in Bcl-X_L_ and MCL-1 mRNA expression was observed in MOLM-13 and THP-1 cells treated with 20(S)-PPD, as shown in Figure 4A.

Subsequently, 20(S)-PPD was investigated for its effect on Bcl-X_L_ and MCL-1 post-transcriptionally. After treating THP-1 and MOLM-13 cells for 8 h with 20(S)-PPD, 10 μg/mL of Cycloheximide (CHX) was used to inhibit the synthesis of proteins. The expressions of Bcl-X_L_ and MCL-1 were then measured at various time points using Western blotting to determine the protein degradation rate and half-life. MCL-1 has been demonstrated to be an unstable protein with a half-life ranging from 1.5 to 4 h, in contrast to Bcl-X_L_, which exhibits stability with a half-life of approximately 20 h (Rooswinkel et al., 2014). Our data indicated that the half-life of MCL-1 in MOLM-13 cells was 131.5 ± 21.1 min, which decreased to 87.7 ± 17 min following the addition of 20(S)-PPD. Similarly, in THP-1 cells, the half-life of MCL-1 was 133.3 ± 5 min, which further decreased to 73.4 ± 9.6 min after the addition of 20(S)-PPD (Figure 4B). Additionally, the half-life of Bcl-X_L_ protein was reduced from 48.2 ± 6.6 h to 26.7 ± 5.8 h in MOLM-13 cells and from 36.1 ± 4.6 h to 21.7 ± 2.3 h in THP-1 cells (Figure 4C). These results indicate that 20(S)-PPD accelerated the Bcl-X_L_ and MCL-1 protein degradation.

To better understand how 20(S)-PPD facilitates the degradation of Bcl-X_L_ and MCL-1, a virtual molecular docking was conducted using AutoDock 4 software. The docking results revealed that 20(S)-PPD directly interacts with either the MCL-1 or Bcl-X_L_ proteins, forming hydrogen bonds with ARG-263 on MCL-1, or with PRO-116 on Bcl-X_L_ (Figures 4D, E). Additionally, 20(S)-PPD exhibited lower Gibbs free energy values of −8.75 kcal/mol and −7.58 kcal/mol for binding to Bcl-X_L_ and MCL-1, respectively (Table 1). These findings suggest that 20(S)-PPD has a strong binding affinity for both proteins. To further elucidate the role of the proteasome in the degradation of Bcl-X_L_ and MCL-1 induced by 20(S)-PPD, we examined the impact of the proteasome inhibitor MG132 on the downregulation of these proteins by 20(S)-PPD. As depicted in Figure 4F, the addition of MG132 significantly mitigated the downregulation of Bcl-X_L_ and MCL-1 by 20(S)-PPD. Collectively, 20(S)-PPD may downregulate MCL-1 or Bcl-X_L_ protein levels by inhibiting transcription and reducing protein stability.

**TABLE 1:** Docking binding energy of 20(S)-PPD to target proteins.

Protein names	Protein ID	Docking binding energy/kcal.mol^−1^
MCL-1	3WIY	−7.58
Bcl-X_L_	3WIZ	−8.75

### 3.5 20(S)-PPD enhanced the anti-leukemic effect of venetoclax on AML cells

**FIGURE 5:**
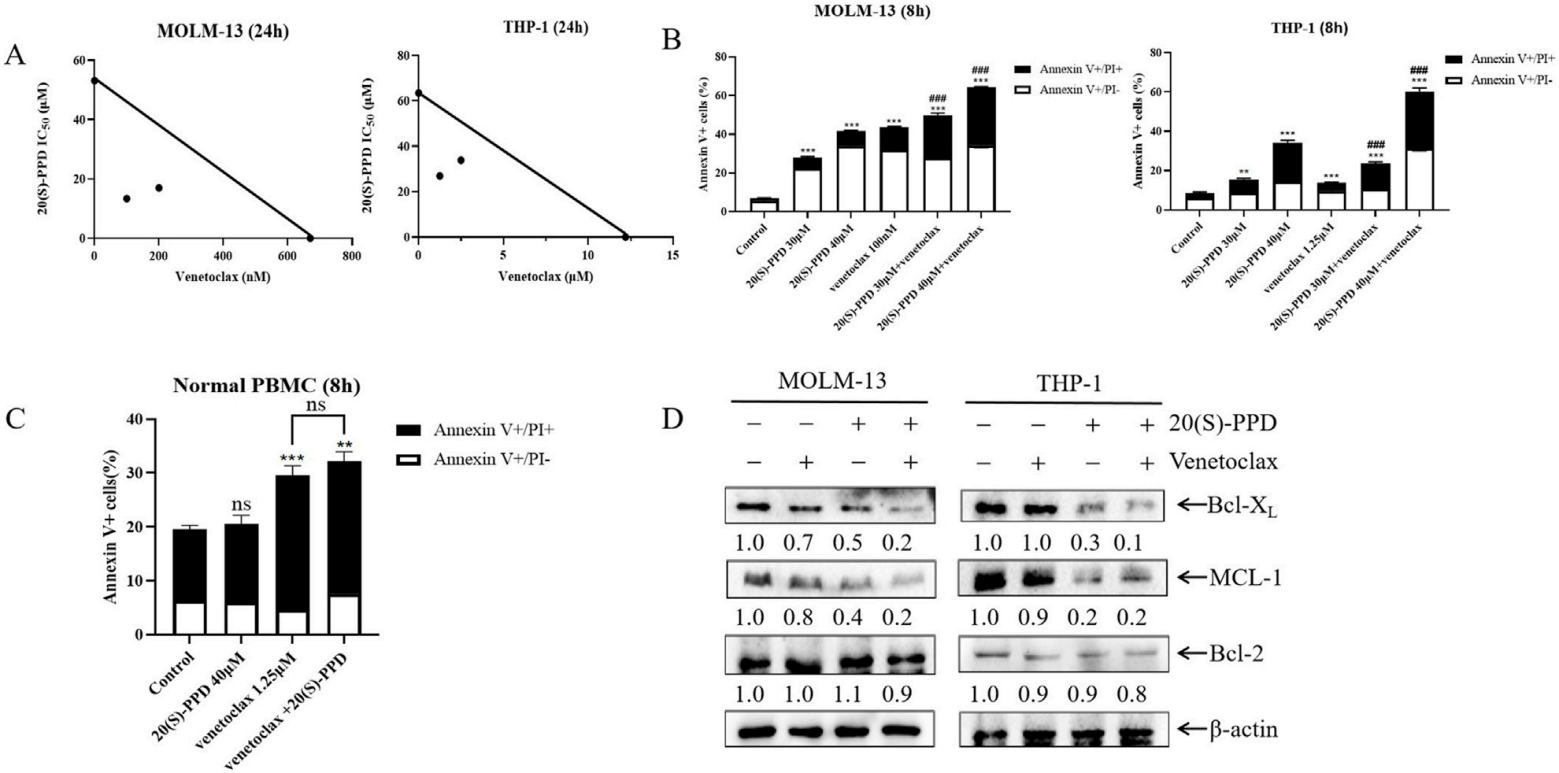
20(S)-PPD enhanced the anti-leukemic effect of venetoclax on AML cells **(A)** MOLM-13 cells (panel left) and THP-1 cells (panel right) were treated with 20(S)-PPD alone or in combination with venetoclax (Bcl-2 inhibitor) for 24 h and then we performed to CCK-8 assay and calculated the IC_50_ values. A plot of the IC_50_ values for each inhibitor appears on the axes; the solid line indicates additive effects, while the points represent concentrations inhibiting proliferation by 50%. Synergistic points are below the line, whereas antagonistic points are above it. **(B)** MOLM-13 cells (panel left) and THP-1 cells (panel right) were treated with 20(S)-PPD alone or in combination with the Bcl-2 inhibitor venetoclax for 8 h and then we performed to Annexin V/PI staining and flow cytometry analyses. **P < 0.01; ***P < 0.001 compared to the no drug treatment control, while ###, P < 0.001 compared to 20(S)-PPD-treated group. **(C)** PBMCs were treated with 40 μM 20(S)-PPD alone or in combination with venetoclax for 8 h and stained with annexin V-FITC/PI. **P < 0.01; ***P < 0.001 compared to the no drug treatment control, while ns, non-significant, compared to venetoclax-treated group. **(D)** MOLM-13 cells (panel left) and THP-1 cells (panel right) were treated with 20(S)-PPD alone or in combination with venetoclax for 8 h and then we performed to WB assay. Densitometry measurements are shown below the corresponding blots, normalized to β-actin and compared to the vehicle control group.

Venetoclax is a selective and potent oral Bcl-2 inhibitor (Guerra et al., 2019). Venetoclax has been used as monotherapy in patients with relapsed/refractory AML but has demonstrated only modest efficacy with an overall response rate (ORR) of 38% (Liu, 2021; Ong et al., 2022). It appears that AML patients have both intrinsic and acquired resistance to venetoclax, resulting in this low ORR. Indeed, due to venetoclax’s specific targeting of Bcl-2, tumor cells may shift their dependency to other anti-apoptotic proteins such as Bcl-X_L_, MCL-1 and Bfl-1 for survival (Shimony et al., 2022). Consequently, this study aims to investigate whether 20(S)-PPD could enhance venetoclax’s sensitivity in treating AML. The CCK-8 assay revealed that the combination of 20(S)-PPD and venetoclax synergistically inhibited THP-1 and MOLM-13 cell proliferation (Figure 5A). Further analysis using Annexin V-FITC/PI staining and flow cytometry indicated that 20(S)-PPD significantly augmented venetoclax-induced apoptosis in THP-1 and MOLM-13 cells (Figure 5B). Additionally, treatment with venetoclax alone (1.25 μM) or in combination with 20(S)-PPD slightly increased the percent of Annexin V-positive cells. However, compared to treatment with venetoclax alone, the combination did not significantly further increase the percent of Annexin V-positive cells (p > 0.05), suggesting that the combination of venetoclax and 20(S)-PPD does not introduce additional toxicity (Figure 5C). Mechanistically, we found that compared to treatment with PPD alone, the combination further downregulated Bcl-X_L_ and MCL-l, although the level of Bcl-2 remained unchanged (Figure 5D). Collectively, these findings indicate that 20(S)-PPD potentiates the anti-leukemic efficacy of venetoclax in AML cells.

## 4 Discussion

The main challenges in treating AML include patient tolerability, susceptibility to drug resistance, and high relapse rates, necessitating the development of novel therapeutic strategies (Limongello et al., 2021; Liu et al., 2022; Sugamori et al., 2022). 20(S)-PPD has demonstrated anti-tumor potential in various solid tumors (Zhu et al., 2011; Zhang et al., 2013; Ben-Eltriki et al., 2016; Zhang et al., 2018; Peng et al., 2019; Lu et al., 2020; Zhang et al., 2020; Fu et al., 2022; Song et al., 2023). This study found that 20(S)-PPD inhibits AML cell proliferation and induces apoptosis (Figures 1A–D). Accumulating evidence suggests that 20(S)-PPD induces apoptosis via the intrinsic/mitochondrial pathway in several preclinical models of both solid tumors and hematological malignancies (Popovich and Kitts, 2002; 2004; Ming et al., 2007). By silencing Bax and Bak, the central effectors of the intrinsic/mitochondrial apoptosis pathway, we demonstrated that 20(S)-PPD-induced apoptosis in AML cells is at least partially mediated through this pathway (Figures 1F, G). Bcl-2 protein family that includes anti-apoptotic members like Bcl-2, MCL1, and Bcl-X_L_, as well as pro-apoptotic members such as BIM, Bax, and Bak, regulates mitochondrial apoptosis (Singh et al., 2019; Carneiro and El-Deiry, 2020). Our data indicate that 20(S)-PPD treatment downregulates the Bcl-X_L_ and MCL-1 expression, while the levels of Bax, Bak, Bcl-2 and Bfl-1 remaind unchanged in three tested AML cells (Figure 2). The expression of Bim exhibited a slight increase in THP-1 and MV4-11 cells, while it remained unchanged in MOLM-13 cells (Figure 2), potentially attributable to the heterogeneity of AML cells.

To elucidate the role of Bcl-X_L_ and MCL-1 in 20(S)-PPD-induced apoptosis, overexpression of Bcl-X_L_ or MCL-1, coupled with the application of selective inhibitors targeting MCL-1 or Bcl-2, provided direct evidence that 20(S)-PPD induces apoptosis in AML cells by inhibiting Bcl-X_L_ and MCL-1 proteins (Figure 3). 20(S)-PPD decreased both mRNA and protein stability of Bcl-X_L_ and MCL-1 in subsequent studies (Figures 4A–C). These findings indicate that the downregulation of Bcl-X_L_ and MCL-1 levels induced by 20(S)-PPD may be achieved through the reduction of their gene expression and the acceleration of protein degradation. Furthermore, our virtual molecular docking analysis suggests that the acceleration of protein degradation by 20(S)-PPD may result from direct interactions between 20(S)-PPD and MCL-1/Bcl-X_L_ molecules (Figures 4D, E). Additionally, a recent study found that 20(S)-PPD induces apoptosis in AML cells by inhibiting the PI3K/AKT/mTOR pathway and the expression of the transcription factor c-Myc (Zhang et al., 2025). Given that the PI3K/AKT/mTOR pathway or c-Myc can regulate the expression of MCL-1 and/or Bcl-X_L_, the downregulation of MCL-1/Bcl-X_L_ by 20(S)-PPD may be mediated by its inhibition of the PI3K/AKT/mTOR pathway or c-Myc expression (Rahmani et al., 2014; Jokinen and Koivunen, 2015; Hu et al., 2020). However, the exact molecular mechanisms still require further investigation.

It is well established that Bcl-X_L_, Bcl-2, and MCL-1 are aberrantly expressed in many cancer cell lines, enabling these cells to circumvent apoptosis (Carter et al., 2020). Several studies have demonstrated that MCL-1 and Bcl-2 are overexpressed frequently in AML cell lines, with high expression levels being associated with poor prognoses and resistance to chemotherapy (Delia et al., 1992; Fowler-Shorten et al., 2024). Through the Gene Expression Omnibus (GEO) database (GSE35008) and Cancer Genome Atlas (TCGA) database, we found that MCL-1 is upregulated in AML patients and negatively correlates with overall survival rates (Supplementary Figures S1A–C). Conversely, the GEO database (GSE35008) and TCGA database analysis indicated that Bcl-X_L_ is downregulated (Supplementary Figures S1A, B). The observation that low expression of Bcl-X_L_ is positively correlated with overall survival rates in early-stage AML patients, but negatively correlated in later stages (Supplementary Figure S1C), is noteworthy. A plausible explanation for this phenomenon is that early-stage AML cells depend on Bcl-X_L_ for survival, whereas late-stage AML cells may rely on other Bcl-2 family members, such as Bcl-2 or MCL-1. This pattern is analogous to findings in T cell development, where early progenitor T cells require Bcl-X_L_ for their development, while more mature T cells depend on Bcl-2 (Ryan et al., 2010; Singh et al., 2019).

A potent Bcl-2 inhibitor, venetoclax, has been approved by the FDA to treat patients with 17p deletions in chronic lymphocytic leukemia (CLL) (Merino et al., 2018; Roberts et al., 2021). As a monotherapy, venetoclax has only achieved a 19% overall response rate (Andreeff et al., 1999; Konopleva et al., 2016). The modest efficacy of venetoclax in AML patients is attributed to intrinsic drug resistance mechanisms. Specifically, the Bcl-X_L_ and/or MCL-1 upregulation represents the primary mechanisms of resistance to venetoclax (Pan et al., 2014; Choudhary et al., 2015; Punnoose et al., 2016; Jayappa et al., 2017; Tahir et al., 2017; Haselager et al., 2020). Preclinical studies have demonstrated that the inhibition of Bcl-X_L_ and/or MCL-1 exhibits significant anti-AML activity and enhances the sensitivity of venetoclax in AML treatment (Carter et al., 2020; Vanna et al., 2020). However, the clinical application of inhibitors targeting Bcl-X_L_ or MCL-1 is constrained by issues of intolerance and potential toxicity. For instance, *in vivo* and *in vitro*, ABT-737 and ABT-263 have shown remarkable anti-tumor activity against various hematological malignancies through inhibition of Bcl-X_L_, Bcl-2, and Bcl-W. Nevertheless, their clinical development is hindered by associated platelet toxicity (Mason et al., 2007; Wilson et al., 2010; Roberts et al., 2016). MCL-1 inhibitors S63845 and AZD5991 exhibit poor tolerance due to their association with myelosuppression and potential cardiac toxicity, respectively (Brennan et al., 2018; Parrondo et al., 2022).

Several studies have indicated that high doses of 20(S)-PPD (100 mg/kg) do not induce weight loss or toxicity such as hepatotoxicity, nephrotoxicity, cardiotoxicity in mice (Zhang et al., 2018; Jo et al., 2021; Fu et al., 2022). These *in vivo* findings suggest that patients may exhibit improved tolerance to 20(S)-PPD. In this study, we observed that even at a concentration of 60 μM, 20(S)-PPD did not increase the proportion of Annexin V+ cells in normal PBMCs (Figure 1E), suggesting a lack of toxicity to normal cells. Since 20(S)-PPD is a dual inhibitor of both Bcl-X_L_ and MCL-1 and exhibits minimal toxicity, it suggests that the combination of 20(S)-PPD and venetoclax may be a potential strategy for treating AML. Indeed, our results demonstrates that 20(S)-PPD synergistically enhances the sensitivity of venetoclax against AML *ex vivo* models (Figure 5). However, further investigations are required to assess the efficacy and toxicity of 20(S)-PPD alone or in combination with venetoclax in AML animal models or clinical trials.

**FIGURE 6:**
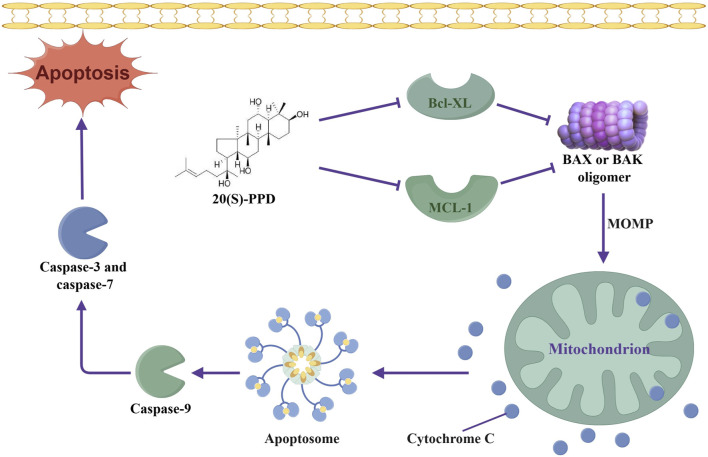
Schematic representation of anti-leukemic mechanism of 20(S)-PPD in AML cells. 20(S)-PPD downregulates Bcl-X_L_ and MCL-1, thereby promoting the oligomerization of Bax/Bak, leading to changes in MOMP, release of cytochrome C, activation of caspase, and ultimately inducing apoptosis. MOMP, mitochondrial membrane permeability.

In conclusion, our results indicate that 20(S)-PPD inhibits proliferation and induces apoptosis in AML cells *in vitro*. Mechanistically, we demonstrate that 20(S)-PPD induces apoptosis in AML cells by down-regulating the protein and transcript levels of Bcl-X_L_ and MCL-1 (Figure 6). Furthermore, our data indicated that 20(S)-PPD synergistically enhances the anti-leukemic activity of venetoclax *in vitro*. The current study suggests that the continued development of 20(S)-PPD as a therapeutic drug for AML would be advantageous.

## Data Availability

The datasets presented in this study can be found in online repositories. The names of the repository/repositories and accession number(s) can be found in the article/Supplementary Material.

## Supplementary material

SUPPLEMENTARY FIGURE S1 The correlation between the gene expression of Bcl-X_L_ and MCL-1 in AML clinical patient sample and overall survival. **(A)** GEO database (GSE35008) analyzed, the gene expression of MCL-1 and Bcl-X_L_ (known as BCL2L1) in AML clinical patient sample and normal sample. G1, AML group; G2, healthy group. **P < 0.01, compared to normal sample; ns, non-significance. **(B)** TCGA database analyzed, the gene expression of MCL-1 and Bcl-X_L_ in AML clinical patient sample and normal sample. TPM, transcripts per million. *P < 0.05, compared to normal sample. **(C)** The association between MCL-1 or Bcl-X_L_ expression and overall survival percent in patients with AML.
