# Supplementary figures and images for: 20(S)-protopanaxadiol inhibits proliferation and induces apoptosis of acute myeloid leukemia cells via targeting Bcl-XL and MCL-1
Source: Front Pharmacol. 2025 Apr 29;16:1530270. doi: 10.3389/fphar.2025.1530270 (PMC12093104; doi:10.3389/fphar.2025.1530270)

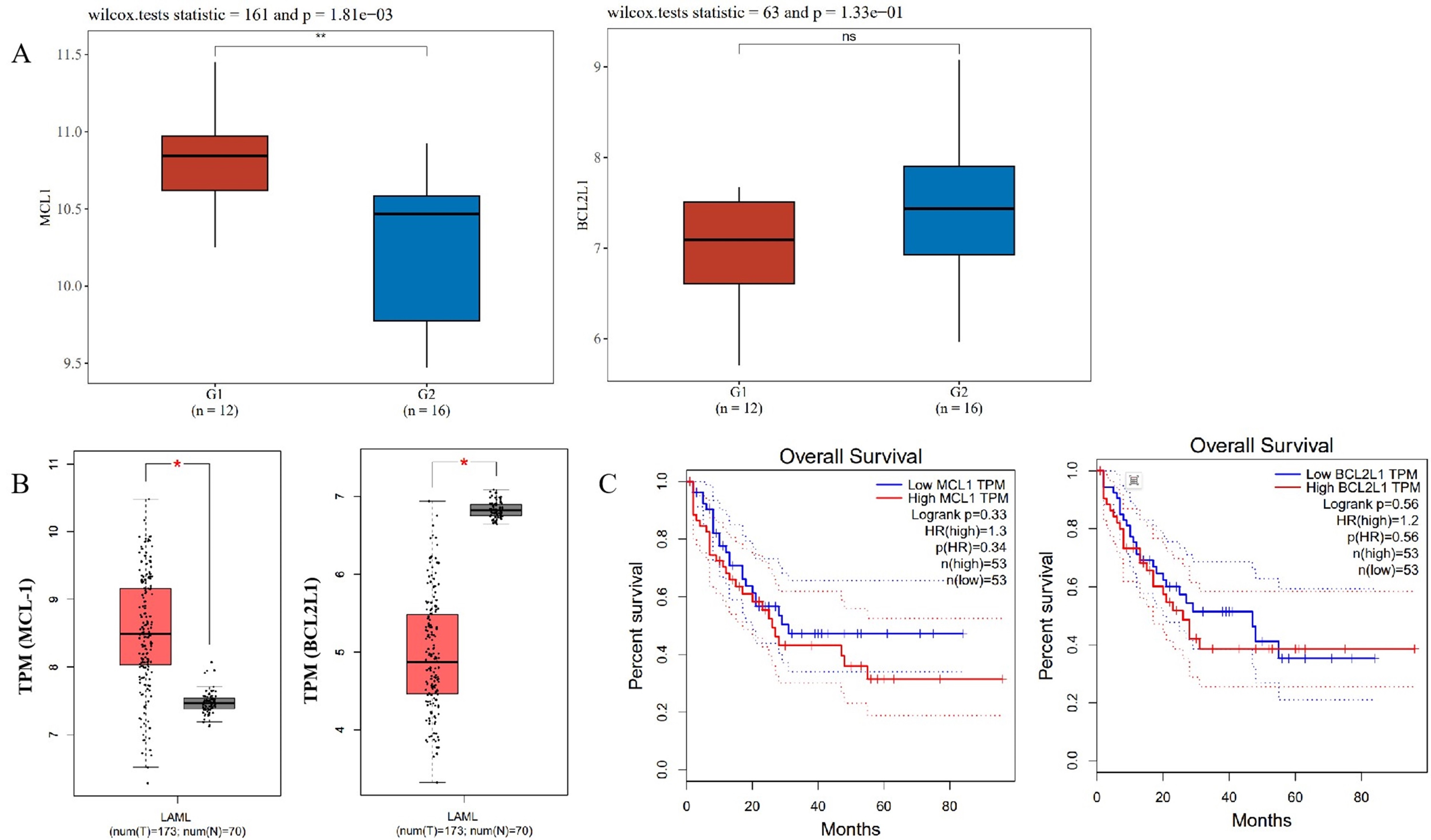

Supplement: Supplementary file 1 [file Image1.jpg]
